# PANoptosis Regulation in Reservoir Hosts of Zoonotic Viruses

**DOI:** 10.3390/v16111733

**Published:** 2024-11-04

**Authors:** Anantika Chandra, Sannula Kesavardhana

**Affiliations:** Department of Biochemistry, Division of Biological Sciences, Indian Institute of Science, Bengaluru 560012, India

**Keywords:** PANoptosis, PANoptosome, inflammation, cell death, innate immunity, reservoir host biology, bats, birds

## Abstract

Zoonotic viruses originating from reservoir hosts, such as bats and birds, often cause severe illness and outbreaks amongst humans. Upon zoonotic virus transmission, infected cells mount innate immune responses that include the activation of programmed cell death pathways to recruit innate immune cells to the site of infection and eliminate viral replication niches. Different inflammatory and non-inflammatory cell death pathways, such as pyroptosis, apoptosis, necroptosis, and PANoptosis can undergo concurrent activation in humans leading to mortality and morbidity during zoonosis. While controlled activation of PANoptosis is vital for viral clearance during infection and restoring tissue homeostasis, uncontrolled PANoptosis activation results in immunopathology during zoonotic virus infections. Intriguingly, animal reservoirs of zoonotic viruses, such as bats and birds, appear to have a unique immune tolerance adaptation, allowing them to host viruses without succumbing to disease. The mechanisms facilitating high viral tolerance in bats and birds are poorly understood. In this perspective review, we discuss the regulation of PANoptotic pathways in bats and birds and indicate how they co-exist with viruses with mild clinical signs and no immunopathology. Understanding the PANoptotic machinery of bats and birds may thus assist us in devising strategies to contain zoonotic outbreaks amongst humans.

## 1. Main Text

Zoonotic viruses are transmitted to humans from animal reservoirs such as bats and birds [1]. In humans, zoonotic viruses cause high morbidity and are often lethal. Zoonotic viruses have caused some of the deadliest recorded pandemics in history. For example, the Spanish Flu pandemic of 1918 and, more recently, the coronavirus disease-19 (COVID-19) pandemic were caused by viral pathogens transmitted from birds and bats, respectively [2]. Bats, in particular, have been proven to be natural reservoirs of several human-infecting zoonotic viruses, such as the severe acute respiratory syndrome coronavirus (SARS-CoV), SARS-CoV-2, Middle East respiratory syndrome coronavirus (MERS-CoV), Ebola, Nipah, Marburg, Hendra viruses, etc. [3,4]. On the other hand, wild aquatic birds are the natural reservoirs of influenza A viruses (IAVs) [5]. 

For successful zoonotic transmission, several factors must align including the rate of viral shedding, environmental persistence of the virus, recipient host susceptibility and permissibility, etc. [6]. Animal viruses preferentially utilize host-receptors for entry into cells. Often times, humans are rendered susceptible to zoonotic viruses due to homology in cellular receptors between themselves and the reservoir host receptors [7]. For instance, bats are known reservoirs of coronaviruses related to the deadly human-infecting SARS-CoV-2. An in-depth analysis of closely related bat coronaviruses such as RaTG13 revealed that it was capable of utilizing the human angiotensin converting enzyme 2 (ACE2) for entry into host cells, indicating possible routes of spillover from bat populations to humans [8]. However, despite entry into cells, zoonotic viruses may not be able to successfully replicate due to lack of supporting host machinery, and/or their inability to evade the host’s immune defenses [9].

Humans tend to be immunologically naïve to zoonotic viruses at the time of transmission. Despite our immune system showing heightened sensing and activation of immune responses, it takes significantly longer to clear out these newly emerged zoonoses [10,11]. During the period between infection with a zoonotic virus and neutralization by the adaptive immune system, the innate immune system controls the infection. To enable robust detection and clearance of viral infections, the innate immune system promotes elimination of infected cells by activating programmed cell death (PCD) pathways [12]. 

PCD can be either inflammatory or non-inflammatory [13]. Inflammatory forms of PCD include pyroptosis and necroptosis, which cause osmotic lysis of infected cells, releasing pathogen-associated molecular patterns (PAMPs), damage-associated molecular patterns (DAMPs), and pro-inflammatory cytokines into the extracellular space [14]. On the other hand, apoptosis is non-lytic and non-inflammatory before it turns into secondary necrosis, and it does not compromise the membrane integrity of the dying cell [15]. 

**Figure 1:**
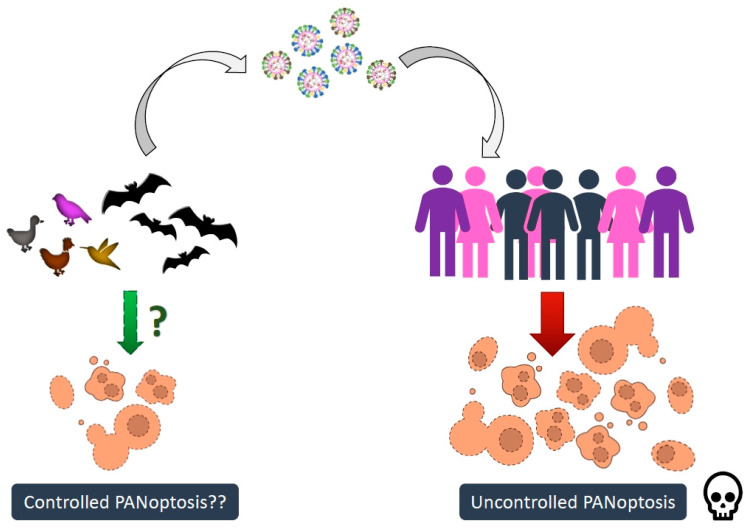
Controlled activation of PANoptosis in bats and birds may assist them in harboring high viral titers without developing clinical symptoms. PANoptosis is a double-edged sword in innate immunity against zoonotic viruses. Controlled activation of PANoptosis during zoonotic virus infection can eliminate viral replication niches and resolve infection. However, uncontrolled activation of PANoptosis during zoonotic virus infection in humans leads to severe morbidity and mortality. Surprisingly, bats and birds demonstrate high immune tolerance to zoonotic viruses without succumbing to disease. The underlying cause for this tolerance may be linked to dampened PANoptosis activation.

Recent biochemical evidence suggests that specific innate immune sensors trigger the activation of a multimodal cell death program called PANoptosis. PANoptosis is defined as a unique lytic inflammatory cell death pathway driven by multi-protein signaling complexes called PANoptosomes [16,17,18]. PANoptosis activation in virus-infected cells promotes inflammatory immune cell migration to the site of infection and clearance of viral replication niches [19]. However, persistent or hyperactivation of PANoptosis can cause tissue damage and overwhelm the body’s repair mechanism, resulting in high morbidity and mortality [20]. Dysregulated inflammation and cell death during H5N1 and SARS-CoV-2 infections are associated with the severity of the disease, indicating PANoptosis as an underlying factor in regulating the priming of pathogenesis. Interestingly, the species serving as reservoirs of zoonotic viruses show mild clinical symptoms and disease progression despite harboring high viral titers, suggesting the possible altered regulation of PANoptosis in them compared to humans. Bats and birds, in particular, harbor pathogenic zoonotic viruses without showing severe pathologies [21,22]. However, the mechanism conferring viral tolerance without hyperactivation of PANoptosis and inflammation in reservoir hosts remains unclear [22]. In this review, we discuss the importance of studying the PANoptotic machinery in bats and birds. We also hypothesize that the divergent evolution of the PANoptotic machinery in bats and birds allows them to tolerate several pathogenic viruses while showing mild immunopathology (Figure 1). 

## 2. PANoptosis and PANoptosome Regulation

PANoptosis is a unique, multimodal cell death pathway executed by large heteromeric complexes called PANoptosomes. Zoonotic pathogens such as IAVs have been found to induce the formation of PANoptosomes at single-cell levels, indicating their role in priming inflammatory cell death pathways during infection [23]. The upstream sensors nucleating PANoptosome complexes are various, including Z-nucleic acid binding protein-1 (ZBP1), absent in melanoma-2 (AIM2), receptor-interacting serine/threonine protein kinase 1 (RIPK1), NOD-like receptor (NLR) family pyrin domain (PYD)-containing 12 (NLRP12) and NLR family caspase activation and recruitment domain (CARD) containing 5 (NLRC5) [16,24,25,26,27]. ZBP1 is a unique Z-RNA sensor that senses Z-RNAs produced during IAV infection, triggering the activation of PANoptosis [28]. Similarly, AIM-2 senses double-stranded DNA molecules produced during herpes simplex virus 1 (HSV1) infection and mediates the formation of PANoptosomes via interaction with ZBP1 [24]. RIPK1 has also been noted to activate PANoptosis in response to *Yersinia* infections leading to significant immunopathology [25]. NLRP12 senses PAMPs along with heme to initiate the activation of PANoptosis in hemolytic diseases [26]. NLRC5 has also been found to respond to PAMPs/DAMPs such as heme followed by association with NLRP12 to drive PANoptosis and inflammatory cell death, highlighting the critical role played by PANoptosomes in a diverse array of immunological insults [27].

PANoptosomes include specific components of all three PCD pathways, forming a multi-faceted macromolecular complex driving the activation of pyroptosis, apoptosis, and necroptosis during immunological disturbances [16]. Although the upstream nucleators may differ, PANoptosomes contain certain shared molecular features, including the presence of ASC (pyroptosis), caspase-8 (CASP8; apoptosis), and RIPK3 (necroptosis) [23]. 

Pyroptosis is a type of inflammatory PCD, initiated by large, cytosolic heterologous oligomeric complexes called inflammasomes. Canonical inflammasomes typically consist of a sensor protein, an adaptor molecule, and pro-caspase 1 [29]. Sensor proteins of inflammasomes such as NLRP1, PYRIN, AIM2, NLRC5, NLRP3, NLRP12 etc. can sense various PAMPs or DAMPs, undergo oligomerization and recruit adaptor proteins to initiate CASP1 activation. For instance, NLRP3 senses bacterial toxins such as nigericin and DAMPs such as ATP, and interacts with the adaptor protein ASC (apoptosis-associated speck-like protein containing a CARD) to generate a heterooligomer which serves as the platform for CASP1 activation [16,28]. Importantly, the sensor proteins instrumental for activating inflammasomes can also activate PANoptosomes [30]. Reciprocally, components of PANoptosomes including RIPK1, CASP8, and the Fas-associated death domain (FADD) can promote the activation of NLRP3 inflammasomes, leading to CASP1 activation [30,31]. Activated CASP1 cleaves Gasdermin D (GSDMD) into an N-terminal domain and a C-terminal fragment [32]. The cleaved N-terminal domain of GSDMD oligomerizes to form pores on the plasma membrane, causing an ionic imbalance and enabling osmotic lysis of the affected cell. Additionally, activated CASP1 cleaves leaderless pro-inflammatory zymogens such as pro-IL-1β and pro-IL-18 into active IL-1β and IL-18 for release through GSDMD pores during cell lysis, further promoting inflammation. 

Apoptosis is a relatively non-lytic form of PCD. Apoptosis can be triggered by either intracellular stress causing the mitochondrial membrane rupture and release of cytochrome C (cytC) into the cytosol (intrinsic apoptosis) or extracellular death ligands (extrinsic apoptosis) [15]. During intrinsic apoptosis, cytC is released into the cytosol, wherein it binds to apoptotic protease activating factor-1 (APAF-1), causing it to oligomerize into a heptameric complex called the apoptosome. The apoptosome serves as a platform for activating the initiator CASP9. Activated CASP9 cleaves and activates CASP3 and CASP7, which act as executioner proteases leading to cell death. Extracellular ligands such as tumor necrosis factor-α (TNFα) can either direct the cell into pro-survival mode or trigger apoptosis by binding to TNF receptor-1 (TNFR1) and initiating the formation of either cytosolic complex I or II, respectively. Complex I, consisting of transforming growth factor-β (TGF-β)-activated kinase 1 (TAK1) and ubiquitinylated RIPK1, directs the cell into survival during stress [33,34]. Interestingly, inhibition of TAK1 leads to RIPK1-dependent activation of PANoptosis, resulting in inflammatory disease [30]. On the other hand, cytosolic complex II, consisting of TNFR1-associated death domain protein (TRADD) and RIPK1, recruits FADD to create a platform for CASP8 activation. Activated CASP8 activates CASP3 and CASP7, leading to extrinsic apoptosis [35]. Additionally, PANoptosomes also provide a platform for CASP8 and RIPK1 interaction leading to apoptosis. Interestingly, CASP8 and CASP3 can also cleave GSDMD and GSDME to induce PANoptosis [36,37]. Therefore, significant crosstalk between the resident molecules of the PANoptosome leads to pan-activation of lytic and non-lytic cell death pathways. 

Inhibition of CASP8 can cause RIPK1 to hetero-oligomerize with RIPK3 via homotypic interactions through their RIP-homotypic interaction motifs (RHIMs) [38,39,40]. The interaction of RIPK1 and RIPK3 leads to the formation of RIPK1-PANoptosomes, which serve as the platform for RIPK3 activation. Activated RIPK3 phosphorylates mixed lineage kinase domain-like pseudokinase (MLKL). Phosphorylated MLKL (pMLKL) oligomerizes to form pores on the plasma membrane, leading to lytic cell death. Also, ZBP1 consists of two RHIM motifs and can interact with RIPK3 to nucleate the formation of PANoptosomes, which serve as platforms for MLKL phosphorylation leading to necroptosis [41,42,43]. 

Regulation of PANoptosomes is critical during viral infection. Controlled activation of PANoptosis during viral infection effectively eliminates viral pathogens. However, hyper-activation of PANoptosis during viral infection causes uncontrolled cell death and is detrimental to the host [44]. Interestingly, bats and birds have appeared to show altered cell death activation, perhaps allowing them to host numerous viruses without harming themselves. The steps determining cell death outcome during PANoptosis are dependent on the sensing/identification of viral PAMPs followed by assembly of the PANoptotic machinery and activation of executioner proteins causing cell death. Therefore, the successful execution of PANoptosis is dependent on the optimal regulation of the sensors and effector molecules of PANoptosis [17]. Possible variations in the sequences of bat, bird, and human PANoptotic components may account for differential regulation of PANoptosis activation, and thus, distinct pathogenesis and disease manifestation outcomes. For instance, the sensor proteins of the human PANoptotic pathway may be more sensitive than those of bats and birds to similar levels of viral PAMPs in the infected cell. Therefore, for similar levels of stimuli, the human PANoptotic pathway may show heightened activation when compared to bats and birds. Conversely, the differences may also be in the expression levels or activation of executioner components of the PANoptotic machinery of bats, birds, and humans. Despite high sensitivity for viral PAMPs, the PANoptotic machinery in bats and birds either show altered expression and activation or have acquired loss-of-function mutations in their executioner proteins (Gasdermins, MLKL, etc.), accounting for dampened cell death during infection. For the list of PANoptosome components taken into consideration in this review, please refer to Table 1.

## 3. The Immune Biology of Reservoir Hosts

Past research in reservoir host biology, particularly bats, has revealed that despite containing conserved homologs of most human immunological pathways, certain components are either uncharacterized or behave differently to their human counterparts. For instance, several bats encode homologs of human pattern recognition receptors (PRRs) such as Toll-like receptors (TLRs), retinoic acid-inducible gene-I (RIG-I), and melanoma differentiation-associated gene 5 (MDA5), stimulator of interferon genes (STING), etc. [46,47,48,49]. Although bat-specific TLR-3, RIG-I, and MDA5 have been demonstrated to hold functional similarity to their human counterparts in terms of exogenous dsRNA detection, their characterization remains incomplete [47,48]. Nevertheless, multiple studies in various species of bats indicate that bats are capable of sensing viral genomic RNA and activating the expression of antiviral innate effector molecules called interferons (IFNs), via the induction of transcription factors such as interferon regulatory factor 3 (IRF3) and IRF7 [47,50,51]. Although species-specific investigation of bats indicates functional conservation of IRF3 and IRF7 in bats, bat IRF7 shows wider tissue distribution and constitutive expression in comparison to humans [51]. Also, despite adaptor proteins responsible for nuclear translocation of IRF3, such as the mitochondrial antiviral-signaling (MAVS) protein, being functionally conserved in certain species of bats, the downstream signaling molecules remain uncharacterized [52].

Notwithstanding the uncharacterized components, it is obvious from the conservation of PRRs, IRFs, and adaptor proteins in bats, that they are capable of viral detection similar to humans and of subsequently activating downstream pathways. This is also emphasized by research demonstrating the retention of functional IFN expression in bats. 

A swift IFN response is critical to limiting viral replication. As such, studies conducted in varying species of bats demonstrate unique modes of IFN up-regulation in bats in comparison to humans. For instance, contraction in IFN gene diversity in black flying foxes is compensated by higher basal level expression of IFNs and interferon-stimulated genes (ISGs) [6,53]. Conversely, Egyptian fruit bats consist of an expanded type I IFN gene bank suggesting finer regulation of ISGs and, hence, viruses [54]. Interestingly, the bat specific homologs of the human 2-5A-dependent endoribonuclease (RNase-L) can be directly stimulated by IFNs instead of depending on intermediate molecules for activation, like their human counterparts [55]. RNase-L is an essential antiviral effector which cleaves viral mRNA to limit infection. Hence, the bat IFN pathways and antiviral effector proteins are differentially regulated in comparison to their human homologs, potentially hinting at the possible reasons behind differential pathological outcomes during zoonosis.

Higher IFN and ISG expression in humans has been linked to excessive inflammation resulting in cell death and mortality during zoonosis [22]. However, despite expressing high levels of IFNs and ISGs, bats appear to control inflammation and induced lytic cell death during viral infection. Effective control of excessive inflammation during viral infection in bats is partially brought about by transcription suppressors (Eg. cRel) of inflammatory cytokines such as TNFα, loss-of-function mutations in STING (a sensor of damaged or double-stranded DNA, which activates the IFN pathway in humans), deletion of the pyrin and HIN domain (PYHIN) genes (critical for microbial DNA sensing and nucleating inflammasomes), etc. [47,49,56,57]. However, attention must be brought to the word “partially” in the previous statement, since a plethora of unexplored and un-reviewed pathways regulating inflammation and cell death in bats remains. 

Similarly, birds also encode functional homologs and orthologs of most human PRRs [58]. In fact, several species of birds have also been found to encode IRFs, and induce IFNs and ISGs in response to various viral infections including highly pathogenic avian influenza (HPAI) viruses [58,59]. However, a lack of comparative studies between human and avian species-specific responses makes it difficult to compare their regulation in their respective species. Presently, we remain unaware of the universal pathways governing the ability of bats and birds to serve as reservoir hosts of deadly human viruses without suffering from disease. More specifically, we are unaware of how bats and birds circumvent inflammatory cell death despite harboring high viral titers. Hyperactivation of PANoptosomes during zoonosis in humans is central to mortality and morbidity in affected patients [20]. As such, it stands to reason that there may be alternate regulatory pathways at play in bats and birds, allowing them to evade the hyperactivation of PANoptosis.

## 4. PANoptosome Machinery Expression and Activation in Bats and Birds

Studies regarding the activation of PCD in bats and birds are emerging and rapidly gaining popularity amongst scientists worldwide. Bats belong to the order *Chiroptera*, which encompasses two suborders—Yangochiroptera and Yinpterochiroptera—and includes approximately 1400 species [60]. Species-specific investigation into bat-mediated regulation of cell death shows altered activation of PCD in comparison to humans [56,61,62,63,64]. A recent study showed that bat primary immune cells, derived from *Pteropus alecto* (*P. alecto*) (Yinpterochiroptera suborder), exhibit reduced activation of the NLRP3 sensor compared to mouse and human cells [61]. Decreased transcriptional priming, bat-specific splice variants, and an evolutionarily divergent leucine-rich repeat (LRR) domain in *P. alecto*-derived NLRP3 led to reduced activation and lower inflammation during RNA virus infection. The presence of the bat-specific splice variant of NLRP3 was also noted in *Myotis davidii* (*M. davidii*) (Yangochiroptera suborder), implying that dampened NLRP3 activation is a strategy employed by multiple bat species in response to infection.

Additionally, studies report the loss of functional AIM2 across ten bat genomes, indicating the loss of AIM2-PANoptosome complexes in these bats [56]. Furthermore, emerging reports suggest that the bat-homolog of human ASC2 is a negative regulator of ASC-dependent cell death complexes in bats [64]. Bat ASC2 showed higher expression and function than human ASC2, and was reported to reduce inflammatory cell death during viral infection. Therefore, bats have dampened NLRP3 activation, lost AIM2 inflammasome expression, and encode potent inhibitors of ASC-dependent cell death complexes to reduce activation of pyroptosis and PANoptosis during viral infection [56,61,64]. *P. alecto* has also been shown to possess inactive CASP1, impairing cleavage and activating pro-inflammatory cytokines such as IL-1β [62]. Interestingly, other bat species of the Yinpterochiroptera suborder, like *Eonycteris spelaean* (*E. spelaean*) and *M. davidii*, express functional CASP1 but continue to exhibit diminished IL-1β cleavage. As such, these bats were found to encode IL-1β with reduced potential for cleavage [62]. Thus, it appears that bats have alternative homologs of CASP1 and IL-1β, causing dampened PANoptosis due to a reduction in IL-1β release post-activation. 

Mutations have also been recorded in GSDMD, a critical executioner of PANoptosis [63]. However, its comprehensive characterization remains to be achieved. Although no studies indicate how GSDMD is regulated in bats, we speculate that bats might have evolved to regulate the GSDMD pore formation and the subsequent activation of cell death, since most of the innate immune pathways converge at GSDMD activation. 

Bats appear to retain ZBP1, RIPK3, RIPK1, and MLKL expression [65,66]. However, whether these molecules regulate PANoptosis activation in bat cells is unclear. We have recently shown that bat cells (Tb1-Lu) express ZBP1 and other RHIM proteins and promote RHIM-protein mediated apoptosis and necroptosis, suggesting the possible operation of PANoptosis in them [65]. Another recent study showed that bats contained higher amino acid substitution rates in ZBP1, RIPK1, RIPK3, and MLKL, indicating rapid evolution [67]. As such, the rapid evolution of innate immune proteins indicates high selection pressure to escape interaction and modulation by pathogen-encoded proteins. Therefore, it appears that the PANoptotic pathway in bats has undergone several adaptations to accommodate viral pathogens without compromising themselves by hyper-activating PANoptosis. However, comprehensive experimental investigation characterizing and comparing the bat-specific and human-specific PANoptotic pathways to demonstrate dampened activation of PANoptosis in bats remains to be carried out. 

Birds belong to the class *Aves*, encompassing more than 11,000 species worldwide [68]. Investigation into the modulation of PANoptosis in birds in the presence of immunological challenge revealed altered effectors and regulation. Recent studies demonstrated the loss of ASC with the concurrent retention of NLRP3 in several species of birds [69]. NLRP3 depends on ASC to generate heterooligomeric platforms for CASP1 activation. Therefore, the loss of ASC with the concurrent retention of NLRP3 points to alternate platforms for CASP1 activation in birds. Indeed, birds encode ASC-independent PANoptosome sensors such as NLRP1 and NLRC4, indicating altered but functional PANoptotic pathways in birds [69]. Also, CASP1 cleavage sites were reported and characterized in bird-encoded GSDMA. While humans encode GSDMA-E and pejvakin, birds are only found to carry genes for GSDMA, GSDME, and pejvakin [70,71]. While no human-encoded caspases have been found to cleave human GSDMA, characterization of chicken GSDMA demonstrated activation of PANoptosis through CASP1 cleavage [71]. Therefore, birds have functional PANoptotic pathways that differ significantly from those of humans.

Although birds are shown to activate non-lytic cell death pathways in response to the influenza A virus challenge, it is unclear whether the molecules driving non-lytic cell death in birds are similar to those in humans [72]. For instance, PANoptosomes can cause non-lytic cell death in humans under certain conditions. However, most of the experiments used to probe for activation of non-lytic cell death pathways in birds have depended on non-specific assays such as the terminal transferase deoxyuridine nick-end labeling (TUNEL) assay due to the absence of chicken-specific antibodies [73,74]. However, genomic databases such as GenBank report genes encoding CASP3, CASP6, CASP7, CASP8, and CASP9 being present in chickens and ducks. Indeed, several groups have depended on available sequences in GenBank for CASP3, CASP6-9, etc., for performing quantitative real-time polymerase chain reaction (qRT-PCR) to show up-regulation of these caspases during non-lytic cell death. For instance, several groups have used qRT-PCR and immunoblotting to show an up-regulation of CASP3 and CASP8 in response to immunological stress [75,76]. Upregulation of cleaved CASP9 has also been demonstrated in tissues undergoing cell death using immunocytochemistry [77]. However, the antibodies used were polyclonal antibodies with reactivity against human CASP9. Further, the study failed to perform qRT-PCR experiments to corroborate the up-regulation of CASP9 in their results. Therefore, when presented with immunological stress, birds appear to express active caspases and undergo cell death. Since CASP8, CASP3, and CASP7 are critical components and executioners of PANoptosis in humans, their activation in birds during cell death indicates active PANoptotic pathways. However, the lack of comprehensive phylogenetic tree maps and comparative enzyme activity assays makes it difficult to draw parallels between the initiators and executioners of PANoptosis in humans and birds.

Phylogenetic analyses on publicly available genomic assemblies and predicted proteomes of several species of birds show conservation of RIPK1 and MLKL [78]. However, the class Aves appeared to have lost expression of ZBP1 and RIPK3 during evolution [78]. Therefore, despite the conservation of RIPK1-PANoptosomes in birds, the loss of ZBP1-PANoptosomes and RIPK3 executioner molecules indicate dampened or alternative pathways for PANoptosis activation in birds. For the list molecular markers, regulators, and executioners of PANoptosis reported in bats and birds, please refer to Table 2 and Table 3 below.

**Table 2:** Summary of the molecular markers, regulators, and executioners of PANoptosis reported in bats.

PANoptosome Component	Abbreviation
Apoptosis-associated speck-like protein containing a caspase-activation and recruitment domain (CARD) domain 2	ASC2 [64]
Caspase-1	CASP1 [62]
Gasdermin D	GSDMD [63]
Interferon regulatory factor-1	IRF1 [66]
Mixed lineage kinase domain-like pseudokinase	MLKL [65,67]
NOD-like receptor (NLR)family CARD-domain containing 5 (NLRC5)	NLRC5 (Not Characterized) [46,48]
NLR-family pyrin domain (PYD)-containing 3	NLRP3 [61]
Receptor-interacting serine/threonine protein kinase 1	RIPK1 [65,67]
Receptor-interacting serine/threonine protein kinase 3	RIPK3 [65,67]
Z-nucleic acid binding protein-1	ZBP1 [65,67]

## 5. Do Bats and Birds Execute PANoptosis?

**Figure 2:**
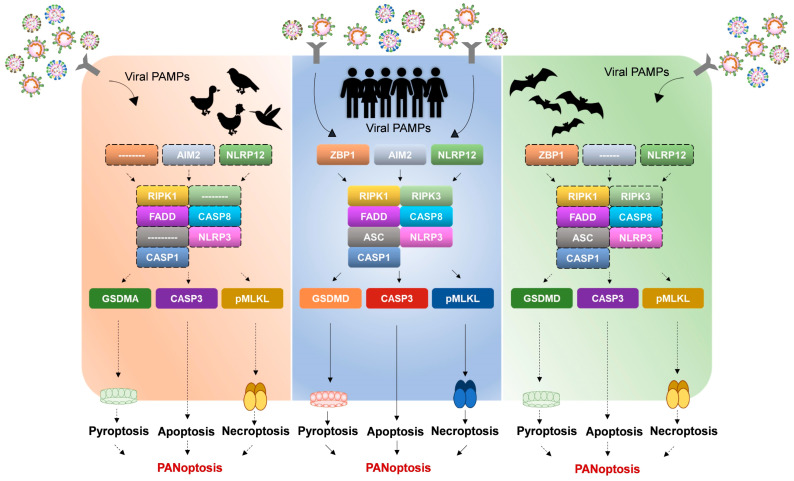
Altered PANoptosomes in birds and bats indicate differential PANoptosis regulation and immune tolerance of zoonotic viruses. The altered composition of PANoptosomes in birds and bats may cause dampened activation of PANoptosis, leading to high immune tolerance for zoonotic viruses in them. Loss of ASC, ZBP1, and RIPK3 expression in birds over time highlights the loss of critical components in bird PANoptosomes. Although bats retain homologs of most of the human PANoptotic machinery, mutations in the critical components of bat-encoded PANoptotic sensors and executioners such as NLRP3 and GSDMD lead to dampened PANoptosis activation. Also, loss of AIM2 expression in bats indicates the deletion of critical viral PAMP sensors in bats, leading to reduced PANoptosis activation. Solid lines and arrows represent PANoptosome pathways activated in humans. Dotted lines and arrows represent loss or dampened activation of PANoptosome components in bats and birds.

Bats and birds appear to retain some, if not all, the molecules necessary for driving PANoptosis (Figure 2). However, comprehensive studies investigating PANoptosis regulation in bats and birds are lacking, and whether they activate PANoptosis during viral infection remains inconclusive. Apart from their role in infection, the PANoptotic machinery has crucial roles in maintaining organismal homeostasis, which has implications in organismal development, tumorigenesis, and autoimmune/inflammatory diseases [80]. It is well-established that due to flight, bats and birds tend to have higher metabolic rates, leading to increased production of reactive oxygen species (ROS) [81,82]. Higher ROS production has historically been linked to cellular damage, death, inflammation, and shorter life spans. However, despite high ROS production, bats and birds have longer life spans [83]. This could be due to an increased expression of oxygen scavengers in bats and birds [84]. However, alterations in the PANoptotic machinery of bats and birds may also result in subversion of excessive inflammatory cell death (Figure 2). For instance, dampened activation of the NLRP3 PANoptosome and loss of double-stranded DNA sensors like AIM2 in bats are strong indicators of adaptations in the PANoptotic machinery of bats to mitigate inflammation-induced damage due to ROS [56,61]. However, the lack of comparative enzyme-activity assays between the PANoptotic pathways of bats and birds makes it difficult to conclude whether all component molecules of PANoptosis undergo dampened activation compared to humans. It may well be that PANoptosis does not exist in bats and birds, and only singular cell death pathways are activated in response to infection. However, that is an improbable scenario, as the principle components of PANoptosomes are present in bats and birds. Therefore, the existence of the component molecules of PANoptosis in bats and birds should be evidence enough for us to consider the activation of PANoptosis in these organisms. 

Between bats and birds, it appears that while bats retain expression of all the critical components driving PANoptosis in humans, birds have lost crucial components of the PANoptosome (Figure 2). For instance, the loss of ZBP1 and RIPK3 in birds not only accounts for additional undiscovered pathways regulating PANptosis in birds but also hints towards their altered regulation [78] (Figure 2). Additionally, the loss of ASC in several species of birds indicates alternative platforms for NLRP3-dependent CASP1 activation, indicative of differences in the molecular composition of bird-encoded and human-encoded PANoptosomes [69]. On the other hand, bats appear to encode homologs of several components of the human PANoptosome machinery. However, bat homologs of the human PANoptosome complex either show reduced activity or are uncharacterized. For instance, bat-NLRP3 has been proven to have reduced activity compared to human-NLRP3, while ZBP1 and RIPK1-mediated PANoptotic pathways are uncharacterized [61]. Therefore, in either host, it appears that the PANoptosome machinery shows dampened activity due to the loss of component molecules, as in birds, or reduced activation in bats (Figure 2). However, comprehensive experimental validation to confirm the same remains to be carried out.

If dampened PANoptosis activation is a true phenomenon in bats and birds, then it is plausible to speculate that it is the underlying cause behind the immunological tolerance in these organisms towards high titers of various viruses. This speculation is supported by the fact that several zoonotic viruses housed by these organisms encode mimics of the human PANoptotic machinery [65,85]. Therefore, it is fair to surmise that zoonoses would have had prior introduction to the PANoptotic machinery in their reservoir hosts to evolve mimics of the molecular components of PANoptosis. Since cell death at early infection points in humans is critical for the elimination of viral replication niches, the ability to regulate the PANoptotic machinery may confer fitness advantages to viruses, allowing their successful zoonotic transmission. However, due to the inherent dampened activity of the PANoptosome in bats and birds, their immunopathological outcome is vastly different to humans despite the harboring of equal viral titers.

## 6. Conclusions

The knowledge gap in our understanding of cell death and inflammation signaling pathways led us to speculate on the possible regulation of the PANoptotic machinery in bats and birds (Figure 1 and Figure 2). Our review summarizes the current knowledge in this field regarding the core components of the PANoptotic pathway in bats and birds. So far, while bats encode homologs of the core components of the human PANoptosome, birds may have either lost expression or developed alternate pathways for activating PANoptosis (Figure 2). Dampened NLRP3 activation, loss of the AIM2 inflammasome, and altered CASP1 activation indicate dampened PANoptosis activation in bats [56,61,62]. Bat-GSDMD homologs also appear to have variations at critical residues, possibly affecting their structure and function [63]. Although experimental characterization of bat-GSDMD molecules is yet to be carried out, we hypothesize that bats may have impaired GSDMD function leading to dampened PANoptosis activation. Additionally, bats have a conserved expression of ZBP1, RIPK3, RIPK1, and MLKL [65,66,67,78,86]. However, their experimental characterization is incomplete. Our recent work indicates that bat-ZBP1 regulates apoptosis and necroptosis, and suggests possible regulation of PANoptosis in bat cells [65]. Hence, it appears that despite encoding homologs of the human PANoptosome, bats may have dampened PANoptosis activation.

On the other hand, birds appear to have lost expression of several PANoptosome components. For instance, birds have lost expression of ASC, ZBP1, and RIPK3 over time [69,78]. Therefore, birds may undergo reduced PANoptosis activation simply due to the loss of the component molecules.

However, most homolog-identification methods depend on sequence-based exploration of complete, well-annotated genomes, which exclude the identification of pseudogenes and novel protein components that cause PANoptosis. It is important to note that bats comprise about 1400 species, whereas birds comprise over 11,000 species worldwide [60,68]. The PANoptotic components described here in this review are based on studies conducted on limited species of bats and birds. For instance, most of the PCD pathway molecules of birds mentioned here are deduced from studies conducted on chickens (*Gallus gallus*) only. However, it is well known that the actual reservoirs of influenza A viruses (a zoonotic pathogen of medical interest) are wild aquatic birds. Similarly, studies of the bat PANoptotic machinery covered in this review are based on limited species of bats. Unfortunately, we suffer from a severe lack of comprehensive genomic data of all wild aquatic birds and bats due to logistical difficulties in sampling. Therefore, it is difficult to conclude whether all bats and birds follow the PANoptotic trends reviewed in this paper.

To manage future pandemic risks, it is crucial to understand the factors influencing the severity of new zoonotic viruses. Unique aspects of reservoir host immunology are speculated to play significant roles in shaping the evolution of viral traits responsible for their ability to infect humans. In this review, we highlight and compare the differences between the PANoptotic machinery in bats and birds with humans, and speculate that the variations between them are the underlying cause for differential disease outcomes in either species.

## Figures and Tables

**Table 1:** Summary of the molecular markers and executioners of PANoptosis in humans [45].

PANoptosome Component	Abbreviation
Absent in melanoma-2	AIM2
Apoptosis-associated speck-like protein containing a caspase-activation and recruitment domain (CARD) domain	ASC
Caspase-1	CASP1
Caspase-3	CASP3
Caspase-4	CASP4
Caspase-5	CASP5
Caspase-6	CASP6
Caspase-7	CASP7
Caspase-8	CASP8
Caspase-9	CASP9
Fas-associated Death Domain	FADD
Gasdermin D	GSDMD
Gasdermin E	GSDME
Interferon regulatory factor-1	IRF1
Mixed lineage kinase domain-like pseudokinase	MLKL
NLR family PYD-containing 12	NLRP12
NLR family CARD-domain containing 5 (NLRC5)	NLRC5
NOD-like receptor (NLR) family pyrin domain (PYD)-containing 3	NLRP3
Receptor-interacting serine/threonine protein kinase 1	RIPK1
Receptor-interacting serine/threonine protein kinase 3	RIPK3
Z-nucleic acid binding protein-1	ZBP1

**Table 3:** Summary of the molecular markers, regulators and executioners of PANoptosis reported in birds.

PANoptosome Component	Abbreviation
Caspase-1	CASP1 [71]
Caspase-3	CASP3 [75]
Caspase-6	CASP6 [79]
Caspase-8	CASP8 [75,76]
Caspase-9	CASP9 [77]
Gasdermin A	GSDMA [71]
Gasdermin E	GSDME [71]
Interferon regulatory factor-1	IRF1 [59]
Mixed lineage kinase domain-like pseudokinase	MLKL [78]
NLR family PYD-containing 12	NLRP12 [69]
NLR family CARD-domain containing 4	NLRC4 [69]
NOD-like receptor (NLR) family pyrin domain (PYD)-containing 1	NLRP1 [69]
NOD-like receptor (NLR) family pyrin domain (PYD)-containing 3	NLRP3 [69]
Receptor-interacting serine/threonine protein kinase 1	RIPK1 [78]

## References

[1] Osorio J.E., Yuill T.M. (2008). Zoonoses. Encyclopedia of Virology.

[2] Agrawal A., Gindodiya A., Deo K., Kashikar S., Fulzele P., Khatib N. (2021). A Comparative Analysis of the Spanish Flu 1918 and COVID-19 Pandemics. Open Public Health J..

[3] Han H.-J., Wen H., Zhou C.-M., Chen F.-F., Luo L.-M., Liu J., Yu X.-J. (2015). Bats as Reservoirs of Severe Emerging Infectious Diseases. Virus Res..

[4] Murphy H.L., Ly H. (2021). Understanding the Prevalence of SARS-CoV-2 (COVID-19) Exposure in Companion, Captive, Wild, and Farmed Animals. Virulence.

[5] Ozawa M., Kawaoka Y. (2013). Cross Talk between Animal and Human Influenza Viruses. Annu. Rev. Anim. Biosci..

[6] Subudhi S., Rapin N., Misra V. (2019). Immune System Modulation and Viral Persistence in Bats: Understanding Viral Spillover. Viruses.

[7] Letko M., Seifert S.N., Olival K.J., Plowright R.K., Munster V.J. (2020). Bat-Borne Virus Diversity, Spillover and Emergence. Nat. Rev. Microbiol..

[8] Shang J., Ye G., Shi K., Wan Y., Luo C., Aihara H., Geng Q., Auerbach A., Li F. (2020). Structural Basis of Receptor Recognition by SARS-CoV-2. Nature.

[9] Escudero-Pérez B., Lalande A., Mathieu C., Lawrence P. (2023). Host-Pathogen Interactions Influencing Zoonotic Spillover Potential and Transmission in Humans. Viruses.

[10] Bhatia R. (2021). Addressing Challenge of Zoonotic Diseases through One Health Approach. Indian J. Med. Res..

[11] Janeway C.A., Travers P., Walport M., Shlomchik M.J. (2001). Principles of Innate and Adaptive Immunity.

[12] Jorgensen I., Rayamajhi M., Miao E.A. (2017). Programmed Cell Death as a Defence against Infection. Nat. Rev. Immunol..

[13] Park W., Wei S., Kim B.-S., Kim B., Bae S.-J., Chae Y.C., Ryu D., Ha K.-T. (2023). Diversity and Complexity of Cell Death: A Historical Review. Exp. Mol. Med..

[14] Galluzzi L., Kepp O., Krautwald S., Kroemer G., Linkermann A. (2014). Molecular Mechanisms of Regulated Necrosis. Semin. Cell Dev. Biol..

[15] Thompson C.B. (1995). Apoptosis in the Pathogenesis and Treatment of Disease. Science.

[16] Malireddi R.K.S., Kesavardhana S., Kanneganti T.-D. (2019). ZBP1 and TAK1: Master Regulators of NLRP3 Inflammasome/Pyroptosis, Apoptosis, and Necroptosis (PAN-Optosis). Front. Cell. Infect. Microbiol..

[17] Christgen S., Zheng M., Kesavardhana S., Karki R., Malireddi R.K.S., Banoth B., Place D.E., Briard B., Sharma B.R., Tuladhar S. (2020). Identification of the PANoptosome: A Molecular Platform Triggering Pyroptosis, Apoptosis, and Necroptosis (PANoptosis). Front. Cell. Infect. Microbiol..

[18] Pandeya A., Kanneganti T.-D. (2024). Therapeutic Potential of PANoptosis: Innate Sensors, Inflammasomes, and RIPKs in PANoptosomes. Trends Mol. Med..

[19] Place D.E., Lee S., Kanneganti T.-D. (2021). PANoptosis in Microbial Infection. Curr. Opin. Microbiol..

[20] Flerlage T., Boyd D.F., Meliopoulos V., Thomas P.G., Schultz-Cherry S. (2021). Influenza Virus and SARS-CoV-2: Pathogenesis and Host Responses in the Respiratory Tract. Nat. Rev. Microbiol..

[21] Weinberg M., Yovel Y. (2022). Revising the Paradigm: Are Bats Really Pathogen Reservoirs or Do They Possess an Efficient Immune System?. iScience.

[22] Mandl J.N., Ahmed R., Barreiro L.B., Daszak P., Epstein J.H., Virgin H.W., Feinberg M.B. (2015). Reservoir Host Immune Responses to Emerging Zoonotic Viruses. Cell.

[23] Wang Y., Pandian N., Han J.-H., Sundaram B., Lee S., Karki R., Guy C.S., Kanneganti T.-D. (2022). Single Cell Analysis of PANoptosome Cell Death Complexes through an Expansion Microscopy Method. Cell. Mol. Life Sci..

[24] Lee S., Karki R., Wang Y., Nguyen L.N., Kalathur R.C., Kanneganti T.-D. (2021). AIM2 Forms a Complex with Pyrin and ZBP1 to Drive PANoptosis and Host Defence. Nature.

[25] Malireddi R.K.S., Kesavardhana S., Karki R., Kancharana B., Burton A.R., Kanneganti T.-D. (2020). RIPK1 Distinctly Regulates Yersinia -Induced Inflammatory Cell Death, PANoptosis. ImmunoHorizons.

[26] Sundaram B., Pandian N., Mall R., Wang Y., Sarkar R., Kim H.J., Malireddi R.K.S., Karki R., Janke L.J., Vogel P. (2023). NLRP12-PANoptosome Activates PANoptosis and Pathology in Response to Heme and PAMPs. Cell.

[27] Sundaram B., Pandian N., Kim H.J., Abdelaal H.M., Mall R., Indari O., Sarkar R., Tweedell R.E., Alonzo E.Q., Klein J. (2024). NLRC5 Senses NAD+ Depletion, Forming a PANoptosome and Driving PANoptosis and Inflammation. Cell.

[28] Kuriakose T., Man S.M., Subbarao Malireddi R.K., Karki R., Kesavardhana S., Place D.E., Neale G., Vogel P., Kanneganti T.-D. (2016). ZBP1/DAI Is an Innate Sensor of Influenza Virus Triggering the NLRP3 Inflammasome and Programmed Cell Death Pathways. Sci. Immunol..

[29] Malik A., Kanneganti T.-D. (2017). Inflammasome Activation and Assembly at a Glance. J. Cell Sci..

[30] Malireddi R.K.S., Gurung P., Kesavardhana S., Samir P., Burton A., Mummareddy H., Vogel P., Pelletier S., Burgula S., Kanneganti T.-D. (2020). Innate Immune Priming in the Absence of TAK1 Drives RIPK1 Kinase Activity–Independent Pyroptosis, Apoptosis, Necroptosis, and Inflammatory Disease. J. Exp. Med..

[31] Gurung P., Anand P.K., Malireddi R.K.S., Vande Walle L., Van Opdenbosch N., Dillon C.P., Weinlich R., Green D.R., Lamkanfi M., Kanneganti T.-D. (2014). FADD and Caspase-8 Mediate Priming and Activation of the Canonical and Noncanonical Nlrp3 Inflammasomes. J. Immunol..

[32] Van Opdenbosch N., Lamkanfi M. (2019). Caspases in Cell Death, Inflammation, and Disease. Immunity.

[33] Kim S.I., Choi M.E. (2012). TGF-β-Activated Kinase-1: New Insights into the Mechanism of TGF-β Signaling and Kidney Disease. Kidney Res. Clin. Pract..

[34] Malireddi R.K.S., Gurung P., Mavuluri J., Dasari T.K., Klco J.M., Chi H., Kanneganti T.-D. (2018). TAK1 Restricts Spontaneous NLRP3 Activation and Cell Death to Control Myeloid Proliferation. J. Exp. Med..

[35] Hsu H., Huang J., Shu H.-B., Baichwal V., Goeddel D.V. (1996). TNF-Dependent Recruitment of the Protein Kinase RIP to the TNF Receptor-1 Signaling Complex. Immunity.

[36] Wang Y., Gao W., Shi X., Ding J., Liu W., He H., Wang K., Shao F. (2017). Chemotherapy Drugs Induce Pyroptosis through Caspase-3 Cleavage of a Gasdermin. Nature.

[37] Sarhan J., Liu B.C., Muendlein H.I., Li P., Nilson R., Tang A.Y., Rongvaux A., Bunnell S.C., Shao F., Green D.R. (2018). Caspase-8 Induces Cleavage of Gasdermin D to Elicit Pyroptosis during Yersinia Infection. Proc. Natl. Acad. Sci. USA.

[38] Van Opdenbosch N., Van Gorp H., Verdonckt M., Saavedra P.H.V., de Vasconcelos N.M., Gonçalves A., Vande Walle L., Demon D., Matusiak M., Van Hauwermeiren F. (2017). Caspase-1 Engagement and TLR-Induced c-FLIP Expression Suppress ASC/Caspase-8-Dependent Apoptosis by Inflammasome Sensors NLRP1b and NLRC4. Cell Rep..

[39] Kaiser W.J., Upton J.W., Long A.B., Livingston-Rosanoff D., Daley-Bauer L.P., Hakem R., Caspary T., Mocarski E.S. (2011). RIP3 Mediates the Embryonic Lethality of Caspase-8-Deficient Mice. Nature.

[40] Zhang H., Zhou X., McQuade T., Li J., Chan F.K.-M., Zhang J. (2011). Functional Complementation between FADD and RIP1 in Embryos and Lymphocytes. Nature.

[41] Rebsamen M., Heinz L.X., Meylan E., Michallet M., Schroder K., Hofmann K., Vazquez J., Benedict C.A., Tschopp J. (2009). DAI/ZBP1 Recruits RIP1 and RIP3 through RIP Homotypic Interaction Motifs to Activate NF-κB. EMBO Rep..

[42] Thapa R.J., Ingram J.P., Ragan K.B., Nogusa S., Boyd D.F., Benitez A.A., Sridharan H., Kosoff R., Shubina M., Landsteiner V.J. (2016). DAI Senses Influenza A Virus Genomic RNA and Activates RIPK3-Dependent Cell Death. Cell Host Microbe.

[43] Basavaraju S., Mishra S., Jindal R., Kesavardhana S. (2022). Emerging Role of ZBP1 in Z-RNA Sensing, Influenza Virus-Induced Cell Death, and Pulmonary Inflammation. MBio.

[44] Nguyen L.N., Kanneganti T.-D. (2022). PANoptosis in Viral Infection: The Missing Puzzle Piece in the Cell Death Field. J. Mol. Biol..

[45] Choudhury S.M., Sarkar R., Karki R., Kanneganti T.-D. (2024). A Comparative Study of Apoptosis, Pyroptosis, Necroptosis, and PANoptosis Components in Mouse and Human Cells. PLoS ONE.

[46] Banerjee A., Baker M.L., Kulcsar K., Misra V., Plowright R., Mossman K. (2020). Novel Insights Into Immune Systems of Bats. Front. Immunol..

[47] Banerjee A., Rapin N., Bollinger T., Misra V. (2017). Lack of Inflammatory Gene Expression in Bats: A Unique Role for a Transcription Repressor. Sci. Rep..

[48] Papenfuss A.T., Baker M.L., Feng Z.-P., Tachedjian M., Crameri G., Cowled C., Ng J., Janardhana V., Field H.E., Wang L.-F. (2012). The Immune Gene Repertoire of an Important Viral Reservoir, the Australian Black Flying Fox. BMC Genom..

[49] Xie J., Li Y., Shen X., Goh G., Zhu Y., Cui J., Wang L.-F., Shi Z.-L., Zhou P. (2018). Dampened STING-Dependent Interferon Activation in Bats. Cell Host Microbe.

[50] Banerjee A., Falzarano D., Rapin N., Lew J., Misra V. (2019). Interferon Regulatory Factor 3-Mediated Signaling Limits Middle-East Respiratory Syndrome (MERS) Coronavirus Propagation in Cells from an Insectivorous Bat. Viruses.

[51] Zhou P., Cowled C., Mansell A., Monaghan P., Green D., Wu L., Shi Z., Wang L.-F., Baker M.L. (2014). IRF7 in the Australian Black Flying Fox, Pteropus Alecto: Evidence for a Unique Expression Pattern and Functional Conservation. PLoS ONE.

[52] Feng H., Sander A.-L., Moreira-Soto A., Yamane D., Drexler J.F., Lemon S.M. (2019). Hepatovirus 3ABC Proteases and Evolution of Mitochondrial Antiviral Signaling Protein (MAVS). J. Hepatol..

[53] Zhou P., Tachedjian M., Wynne J.W., Boyd V., Cui J., Smith I., Cowled C., Ng J.H.J., Mok L., Michalski W.P. (2016). Contraction of the Type I IFN Locus and Unusual Constitutive Expression of IFN-α in Bats. Proc. Natl. Acad. Sci. USA.

[54] Pavlovich S.S., Lovett S.P., Koroleva G., Guito J.C., Arnold C.E., Nagle E.R., Kulcsar K., Lee A., Thibaud-Nissen F., Hume A.J. (2018). The Egyptian Rousette Genome Reveals Unexpected Features of Bat Antiviral Immunity. Cell.

[55] De La Cruz-Rivera P.C., Kanchwala M., Liang H., Kumar A., Wang L.-F., Xing C., Schoggins J.W. (2018). The IFN Response in Bats Displays Distinctive IFN-Stimulated Gene Expression Kinetics with Atypical RNASEL Induction. J. Immunol..

[56] Ahn M., Cui J., Irving A.T., Wang L.-F. (2016). Unique Loss of the PYHIN Gene Family in Bats Amongst Mammals: Implications for Inflammasome Sensing. Sci. Rep..

[57] Zhang G., Cowled C., Shi Z., Huang Z., Bishop-Lilly K.A., Fang X., Wynne J.W., Xiong Z., Baker M.L., Zhao W. (2013). Comparative Analysis of Bat Genomes Provides Insight into the Evolution of Flight and Immunity. Science.

[58] Chen S., Cheng A., Wang M. (2013). Innate Sensing of Viruses by Pattern Recognition Receptors in Birds. Vet. Res..

[59] Xiang C., Yang Z., Xiong T., Wang T., Yang J., Huang M., Liu D., Chen R. (2022). Avian IRF1 and IRF7 Play Overlapping and Distinct Roles in Regulating IFN-Dependent and -Independent Antiviral Responses to Duck Tembusu Virus Infection. Viruses.

[60] Springer M.S. (2013). Phylogenetics: Bats United, Microbats Divided. Curr. Biol..

[61] Ahn M., Anderson D.E., Zhang Q., Tan C.W., Lim B.L., Luko K., Wen M., Chia W.N., Mani S., Wang L.C. (2019). Dampened NLRP3-Mediated Inflammation in Bats and Implications for a Special Viral Reservoir Host. Nat. Microbiol..

[62] Goh G., Ahn M., Zhu F., Lee L.B., Luo D., Irving A.T., Wang L.-F. (2020). Complementary Regulation of Caspase-1 and IL-1β Reveals Additional Mechanisms of Dampened Inflammation in Bats. Proc. Natl. Acad. Sci. USA.

[63] Nagaraja S., Jain D., Kesavardhana S. (2022). Inflammasome Regulation in Driving COVID-19 Severity in Humans and Immune Tolerance in Bats. J. Leukoc. Biol..

[64] Ahn M., Chen V.C.W., Rozario P., Ng W.L., Kong P.S., Sia W.R., Kang A.E.Z., Su Q., Nguyen L.H., Zhu F. (2023). Bat ASC2 Suppresses Inflammasomes and Ameliorates Inflammatory Diseases. Cell.

[65] Mishra S., Jain D., Dey A.A., Nagaraja S., Khatun O., Balamurugan K., Anand M., Ganji M., Kesavardhana S. (2024). Bat RNA Viruses Employ Viral RHIMs Orchestrating Species-Specific Cell Death Programs Linked to Z-RNA Sensing and ZBP1-RIPK3 Signaling. bioRxiv.

[66] Irving A.T., Zhang Q., Kong P.-S., Luko K., Rozario P., Wen M., Zhu F., Zhou P., Ng J.H.J., Sobota R.M. (2020). Interferon Regulatory Factors IRF1 and IRF7 Directly Regulate Gene Expression in Bats in Response to Viral Infection. Cell Rep..

[67] Palmer S.N., Chappidi S., Pinkham C., Hancks D.C. (2021). Evolutionary Profile for (Host and Viral) MLKL Indicates Its Activities as a Battlefront for Extensive Counteradaptation. Mol. Biol. Evol..

[68] Lees A.C., Haskell L., Allinson T., Bezeng S.B., Burfield I.J., Renjifo L.M., Rosenberg K.V., Viswanathan A., Butchart S.H.M. (2022). State of the World’s Birds. Annu. Rev. Environ. Resour..

[69] Billman Z.P., Hancks D.C., Miao E.A. (2024). Unanticipated Loss of Inflammasomes in Birds. Genome Biol. Evol..

[70] Broz P., Pelegrín P., Shao F. (2020). The Gasdermins, a Protein Family Executing Cell Death and Inflammation. Nat. Rev. Immunol..

[71] Liu J., Wang X., Wang X., Wang J., Ma Y., Cao Y., Zhang W. (2024). Chicken Gasdermins Mediate Pyroptosis after the Cleavage by Caspases. Int. J. Biol. Macromol..

[72] Vasconcelos A.C. Expression of VP2, Caspase 3 and Caspase 8 genes in IBDV infected chicks. Proceedings of the Western Poultry Disease Conference.

[73] Lin H.Y., Chuang S.T., Chen Y.T., Shih W.L., Chang C.D., Liu H.J. (2007). Avian Reovirus-Induced Apoptosis Related to Tissue Injury. Avian Pathol..

[74] Ito T., Kobayashi Y., Morita T., Horimoto T., Kawaoka Y. (2002). Virulent Influenza A Viruses Induce Apoptosis in Chickens. Virus Res..

[75] Miao Z., Miao Z., Shi X., Wu H., Yao Y., Xu S. (2022). The Antagonistic Effect of Selenium on Lead-Induced Apoptosis and Necroptosis via P38/JNK/ERK Pathway in Chicken Kidney. Ecotoxicol. Environ. Saf..

[76] Zhirong Z., Qiaojian Z., Chunjing X., Shengchen W., Jiahe L., Zhaoyi L., Shu L. (2021). Methionine Selenium Antagonizes LPS-induced Necroptosis in the Chicken Liver via the MiR-155/TRAF3/MAPK Axis. J. Cell. Physiol..

[77] Wilkinson B., Elam J., Fadool D., Hyson R. (2003). Afferent Regulation of Cytochrome-c and Active Caspase-9 in the Avian Cochlear Nucleus. Neuroscience.

[78] Dondelinger Y., Hulpiau P., Saeys Y., Bertrand M.J.M., Vandenabeele P. (2016). An Evolutionary Perspective on the Necroptotic Pathway. Trends Cell Biol..

[79] Johnson A.L., Bridgham J.T. (2000). Caspase-3 and -6 Expression and Enzyme Activity in Hen Granulosa Cells1. Biol. Reprod..

[80] Samir P., Malireddi R.K.S., Kanneganti T.-D. (2020). The PANoptosome: A Deadly Protein Complex Driving Pyroptosis, Apoptosis, and Necroptosis (PANoptosis). Front. Cell. Infect. Microbiol..

[81] Castiglione G.M., Xu Z., Zhou L., Duh E.J. (2020). Adaptation of the Master Antioxidant Response Connects Metabolism, Lifespan and Feather Development Pathways in Birds. Nat. Commun..

[82] Pereira F.D., Mena Canata D.A., Salomon T.B., Hackenhaar F.S., Pereira M.J.R., Benfato M.S., Rampelotto P.H. (2023). Oxidative Stress and Antioxidant Defense in the Heart, Liver, and Kidney of Bat Species with Different Feeding Habits. Int. J. Mol. Sci..

[83] Munshi-South J., Wilkinson G.S. (2010). Bats and Birds: Exceptional Longevity despite High Metabolic Rates. Ageing Res. Rev..

[84] Hickey A.J.R., Jüllig M., Aitken J., Loomes K., Hauber M.E., Phillips A.R.J. (2012). Birds and Longevity: Does Flight Driven Aerobicity Provide an Oxidative Sink?. Ageing Res. Rev..

[85] Danthi P. (2016). Viruses and the Diversity of Cell Death. Annu. Rev. Virol..

[86] Águeda-Pinto A., Alves L.Q., Neves F., McFadden G., Jacobs B.L., Castro L.F.C., Rahman M.M., Esteves P.J. (2021). Convergent Loss of the Necroptosis Pathway in Disparate Mammalian Lineages Shapes Viruses Countermeasures. Front. Immunol..

